# The Prevalence of Attention Deficit Hyperactivity Disorder in Psychotic Disorders: Systematic Review and Meta-analysis

**DOI:** 10.1093/schbul/sbae228

**Published:** 2025-01-13

**Authors:** Nicholas Cheng, Shayden Bryce, Michael Takagi, Allie Pert, Audrey Rattray, Evangeline Fisher, Marcus Lai, Mia Geljic, Sarah Youn, Stephen J Wood, Kelly Allott

**Affiliations:** Turner Institute for Brain and Mental Health, Monash University, Clayton, Victoria 3168, Australia; Orygen, Parkville, Victoria 3052, Australia; Centre for Youth Mental Health, The University of Melbourne, Parkville, Victoria 3052, Australia; Orygen, Parkville, Victoria 3052, Australia; Centre for Youth Mental Health, The University of Melbourne, Parkville, Victoria 3052, Australia; Alfred Mental and Addiction Health, Melbourne, Victoria 3004, Australia; Turner Institute for Brain and Mental Health, Monash University, Clayton, Victoria 3168, Australia; Murdoch Children’s Research Institute, Parkville, Victoria 3052, Australia; Orygen, Parkville, Victoria 3052, Australia; Deakin University, Burwood, Victoria 3125, Australia; Orygen, Parkville, Victoria 3052, Australia; Centre for Youth Mental Health, The University of Melbourne, Parkville, Victoria 3052, Australia; Orygen, Parkville, Victoria 3052, Australia; Centre for Youth Mental Health, The University of Melbourne, Parkville, Victoria 3052, Australia; School of Psychological Sciences, The University of Melbourne, Parkville, Victoria 3004, Australia; Orygen, Parkville, Victoria 3052, Australia; Centre for Youth Mental Health, The University of Melbourne, Parkville, Victoria 3052, Australia; Deakin University, Burwood, Victoria 3125, Australia; Orygen, Parkville, Victoria 3052, Australia; Centre for Youth Mental Health, The University of Melbourne, Parkville, Victoria 3052, Australia; Deakin University, Burwood, Victoria 3125, Australia; Orygen, Parkville, Victoria 3052, Australia; Centre for Youth Mental Health, The University of Melbourne, Parkville, Victoria 3052, Australia; School of Psychology, University of Birmingham, Edgbaston, Birmingham B15 2TT, United Kingdom; Orygen, Parkville, Victoria 3052, Australia; Centre for Youth Mental Health, The University of Melbourne, Parkville, Victoria 3052, Australia; School of Psychological Sciences, The University of Melbourne, Parkville, Victoria 3004, Australia

**Keywords:** schizophrenia, attention deficit disorder, comorbidity, neurodevelopmental disorders, psychosis

## Abstract

**Background:**

Although attention deficit hyperactivity disorder (ADHD) is known to be common in psychotic disorders, reported prevalence rates vary widely, with limited understanding of how different factors (eg, assessment methods, geographical region) may be associated with this variation. The aim was to conduct a systematic review and meta-analysis to determine the prevalence of ADHD in psychotic disorders and factors associated with the variability in reported rates.

**Study Design:**

Searches were conducted in MEDLINE, Embase, PsycINFO, CINAHL, and Scopus in May 2023. Studies were eligible if the frequency of ADHD was reported in psychotic disorder samples. Pooled prevalence meta-analyses were performed. Subgroup analyses and meta-regressions explored whether demographic and study characteristics were associated with reported rates.

**Study Results:**

Thirty-six studies were included, involving 30 726 individuals. The pooled lifetime prevalence of ADHD in psychotic disorders was 18.49% (95% CI 11.78%, 27.83%). The between-study heterogeneity was high (*I*^2^ = 98.4% [95% CI 98.2%, 98.6%]). Subgroup analyses revealed higher prevalence rates when using ADHD DSM-IV criteria compared to International Classification of Diseases (ICD)-10. Rates in childhood-onset psychotic disorders were higher than adolescent- and adult-onset psychotic disorder samples. Rates were higher in North America compared to other regions. Meta-regressions indicated a decrease in prevalence rates with publication year.

**Conclusions:**

The prevalence of ADHD in psychotic disorders appears higher than in the general population, highlighting the need for clinical attention and further research into this comorbidity. Reported rates, however, vary significantly. Reasons may include diagnostic criteria, age of psychosis onset, region, study design, and publication year. Future research should investigate these factors using rigorous ADHD assessment protocols.

## Introduction

Attention deficit hyperactivity disorder (ADHD) is a neurodevelopmental condition estimated to affect 5%-8% of children and adolescents, and 2%-6% of adults globally.^1–3^ Individuals with ADHD experience difficulties in directing attention, hyperactivity, and impulsivity that are disproportionate to their age-related peers, disrupting their ability to function and participate in the community.^1^ Attention deficit hyperactivity disorder appears to be particularly prevalent among various psychiatric disorders, including psychotic disorders.^4–8^

Multiple theories have been proposed to explain the relationship between psychotic disorders and ADHD. One theory suggests that individuals with ADHD are at an elevated risk of developing psychotic disorders due to shared neurodevelopmental and environmental factors, including shared polygenic risk, and perinatal complications.^9–11^ For example, a meta-analysis by Nourredine et al.^11^ found that individuals with childhood ADHD have a higher relative risk of developing a subsequent psychotic disorder compared to those without childhood ADHD. Additionally, individuals with a first-degree family history of ADHD are at a higher risk of developing schizophrenia compared to the general population.^12^ Neurobiologically, both disorders are linked to dopaminergic dysregulation in the mesolimbic and mesocortical pathways.^13–15^ This dysregulation has led to theories suggesting that disruptions in reward systems contribute to trait-based impulsivity and increased substance use, which may mediate the relationship between ADHD and psychosis.^5,16–18^ Moreover, there is mixed evidence suggesting that amphetamine-based psychostimulants prescribed for ADHD may contribute to the development of positive psychotic symptoms and subsequent psychotic disorders.^19–21^ Lastly, the cognitive impairments commonly observed in both ADHD and psychotic disorders, such as deficits in attention, working memory, and executive function, may contribute to diagnostic overlap, where premorbid cognitive symptoms of psychosis are interpreted as a presentation of ADHD.^22–24^ This diagnostic overlap may occur during the clinical high risk (CHR) for psychosis state, where subthreshold symptoms are nonspecific, varied, and may resemble ADHD symptoms before a transition to a full-threshold psychotic disorder.^25^

Although the association between ADHD and psychotic disorders is well-established, including evidence of an elevated risk of psychotic disorders among individuals with ADHD,^11^ the extent of the commonality remains underexplored. An analysis including cross-sectional studies and adult ADHD diagnoses, which can retrospectively identify cases missed in childhood and therefore not captured by longitudinal studies of risk, has yet to be performed. This is an important area for further investigation, as understanding the prevalence rate could inform and support awareness among clinicians working with people with psychotic disorders.

Although most reported prevalence rates of ADHD among people with psychotic disorders are higher than general population estimates, they vary widely. One systematic review reported ADHD prevalence in schizophrenia ranged from 17% to 57% during childhood (<18 years old) and from 10% to 47% in adulthood.^26^ The prevalence of ADHD is also notably high in first-episode psychosis (FEP) samples, but again with wide variation. For example, a study conducted at a Spanish early intervention psychosis service found that 24% of individuals with FEP also had a comorbid DSM-IV diagnosis of ADHD.^27^ Additionally, around 15% of individuals with FEP in a Canadian specialized early intervention clinic had ADHD.^28^ However, a Swedish nationwide study of individuals aged 16–25 experiencing their FEP reported an ADHD prevalence of 8.1%, which overlaps with the upper end of the general population estimates for children and adolescents, at 5%-8%.^5^ Attention deficit hyperactivity disorder prevalence also appears to be particularly high in childhood-onset psychotic disorders (COP; ie, psychotic disorders developing before 13 years of age), with rates in study samples ranging between 25% and 84%.^29–31^

Therefore, although the general consensus is that ADHD is commonly observed in psychotic disorders, there is substantial variation in prevalence estimates. This variability mirrors that seen in general population studies, which suggest that the high variability in reported prevalence is likely driven by differences in ADHD assessment methods and study designs, including diagnostic criteria (eg, DSM vs International Classification of Diseases [ICD]), assessment modes (eg, self-report vs interview), sources of information (eg, teacher vs parent), and sample sources and sizes.^3,32,33^ For example, 1 meta-analysis identified lower prevalence rates of ADHD in samples assessed using ICD-10 criteria compared to those using DSM-IV, suggesting that this may be due to the ICD-10’s more stringent requirements for hyperactivity/impulsivity symptoms in diagnosis.^3^ Moreover, socio-culturally influenced factors such as access to healthcare, diagnostic incentives, and the training of healthcare professionals, may contribute to geographical differences in reported prevalence rates in the general population, though evidence showing regional differences is mixed.^34–36^ Some studies have shown evidence of ADHD being more prevalent in North America than in the rest of the world, suggesting a regional bias.^33,37^ However, other studies find consistent rates across regions, indicating a uniform expression of ADHD across cultures.^3,38^

The aim of this study was to systematically review the literature on the prevalence of ADHD in psychotic disorders, determine the overall pooled prevalence estimate, and investigate the factors associated with variation in prevalence rates.

## Methods

### Protocol Registration and Standards

This review was prospectively registered with the international prospective register of systematic reviews (PROSPERO; CRD42023426520) and was conducted in accordance with the Preferred Reporting Items for Systematic Review and Meta-Analysis (PRISMA) guidelines.^39^ The review also met AMSTAR (ie, quality of conduct) criteria where applicable, addressing several methodological shortcomings in previous reviews of psychosis (eg, protocol registration, assessment of publication bias).^40,41^

### Search Strategy

A comprehensive search undertaken in April 2023 on protocol registers (PROSPERO, Open Science Framework, and JBI) showed that there were no ongoing or completed reviews investigating the prevalence of ADHD in individuals diagnosed with a psychotic disorder. Search terms and databases were selected under the guidance of senior researchers and consultation with a Monash University librarian specialized in medical, nursing, and health sciences research. The key terms ([psychosis OR psychoses OR psychotic disorder* OR schizo*] AND [ADHD OR attention deficit disorder with hyperactivity OR attention deficit hyperactivity disorder OR attention deficit disorder] AND [prevalence* OR comorbid*]) were searched in 5 databases (MEDLINE, Embase, PsycINFO, CINAHL, and Scopus) in May 2023. No limits were applied.

### Eligibility Criteria

#### Participants

Studies were considered for inclusion if participants met the criteria for a psychotic disorder according to the Diagnostic and Statistical Manual of Mental Disorders (DSM)^42^ or the ICD.^43^ This included diagnoses such as schizophrenia, schizoaffective disorder, schizophreniform disorder, substance-induced psychotic disorder, delusional disorder, brief psychotic disorder, or unspecified psychotic disorder. Confirmation of psychotic disorder diagnoses was required using established and standardized diagnostic tools such as the Structured Clinical Interview for the DSM (SCID)^44^ or the Kiddie Schedule for Affective Disorders and Schizophrenia (K-SADS).^45^

Exclusion criteria were applied to studies where the proportion or frequency of ADHD was reported in a sample where the psychotic disorder is secondary to another condition (eg, autism spectrum disorder, DiGeorge [22q11.2 deletion] syndrome, schizotypal disorder, bipolar disorder, and post-traumatic stress disorder). Studies focusing on these conditions were considered only if there was a distinct group of participants with a psychotic disorder. Studies were also excluded if they defined psychotic disorders as, or the psychotic group comprised only of, individuals with subthreshold/attenuated psychosis or psychotic-like symptoms. This population was excluded as it represents a distinct clinical group that is highly heterogeneous, with a wide range of symptom profiles and subtypes. Additionally, only 20%-30% of individuals with subthreshold or attenuated psychosis progress to a full-threshold psychotic disorder,^46,47^ meaning that including this population may confound the interpretation of prevalence rates in this study.

#### Variables of Interest

The variables of interest included: (1) the frequency and proportion of a current or lifetime ADHD diagnosis in individuals diagnosed with a psychotic disorder, and (2) the frequency and proportion of individuals deemed to have ADHD currently or during their lifetime according to a cutoff score on a severity scale.

#### Types of Studies

Only peer-reviewed and English-language studies were included. Register, cross-sectional, and longitudinal studies reporting baseline data on the frequency of ADHD in psychotic disorders were also considered. Baseline data from longitudinal studies and randomized controlled trial studies were used to maximize the sample size. To maximize the number of studies included, there was no required minimum sample size. Animal studies, review articles, case studies, conference abstracts, and dissertations were excluded.

### Study Selection

The study selection process consisted of 4 phases. In phase 1, N.C. conducted the initial searches, and N.C., A.P., A.R., E.F., M.G., and S.Y. performed title and abstract screening. Each title and abstract were reviewed by 2 authors, with conflicts resolved by a third party who reviewed the title and abstract independently. In phase 2, N.C., A.P., A.R., E.F., M.G., and S.Y. independently screened the full texts against eligibility criteria. Each full text was reviewed by 2 authors. Conflicts were resolved with a third party and via discussions with senior authors if the decision remained uncertain. Authors of studies that met all other inclusion criteria but did not report the prevalence or frequency of ADHD within psychotic disorders (eg, studies reporting the presence of comorbidities but separated prevalence or frequency data for psychotic disorders and ADHD) were contacted for additional data. Phase 3 involved searching the reference lists of relevant reviews for additional articles that might meet the inclusion criteria. The final phase involved identifying and removing overlapping samples across studies.

### Data Extraction

Authors N.C. and M.L. independently extracted data from all included studies using a custom extraction form. Information extracted from the studies included: mean age, year of publication, gender identity (% female), ADHD diagnostic criteria, region (North America; Europe; Asia; Africa; Middle East; Oceania; South America), study design (Register; Medical Records; Cohort; Cross-sectional; Other), study setting (Clinical; Non-clinical), type of ADHD assessment (Interview; Self-Report; Mixed), and informant (eg, caregiver, teacher) involvement in ADHD assessment (Informant; No Informant). For the type of psychotic disorder, study samples were allocated as either schizophrenia spectrum or non-schizophrenia spectrum if 80% of the sample comprised of one of these categories and “Unclear” if the type was not clearly stated. Any discrepancies were resolved via discussion. We contacted 46 authors for additional data and received replies from 5.^48–52^

### Appraisal of Methodological Quality

Each study’s methodological quality was evaluated using a 5-item scale adapted from the Newcastle-Ottawa quality assessment scale^53^ (see Supplementary Material). Scores of 2, 1, or 0 were assigned to each item, with a maximum possible total score of 10 indicating higher quality. Authors N.C. and M.L. independently assessed the risk of bias for each study. Any disagreements were resolved by a third author, A.P., via independent rating.

### Statistical Analysis

Statistical analyses were conducted using *R* Version 4.2.2. Meta-analyses were performed with the *meta* and *dmetar* packages.^54,55^ A generalized mixed-effects model was used to calculate the pooled lifetime prevalence of ADHD in psychotic disorders. Sensitivity analyses of the pooled prevalence rates were conducted by identifying and excluding outliers (ie, studies where the 95% CI of the estimated prevalence does not overlap with the 95% CI of the overall pooled prevalence)^56^ and repeating the analyses with studies with *n* ≥ 10 and *n* ≥ 20 participants in the sample. Between-study heterogeneity was examined with the *I*^2^ statistic. Because meta-analyses tend to produce high *I*^2^ statistics, we also report the prediction interval, which gives an estimate of the plausible range of prevalence rates where a new study is expected to lie.^57,58^

Subgroup analyses were conducted to determine the pooled prevalence of ADHD according to the region (North America; Europe; Other), study design (Register; Medical Records; Cohort; Cross-sectional; Other), study setting (Clinical; Non-Clinical), type of ADHD assessment (Interview; Self-Report; Other; Unclear), ADHD diagnostic criteria (DSM-IV; DSM-5; ICD-10), informant involvement in ADHD assessment (Informant Involved; No Informant; Unclear), and type of psychotic disorder (Schizophrenia Spectrum Disorder; Other Psychotic Disorder; Unclear). Between-group differences in the logit-transformed pooled prevalence were calculated using the *Q* statistic. For analyses with 3 or more subgroups and with significant between-groups differences, pairwise comparisons were performed by conducting a *z-*test to determine where the difference lay.^59,60^ Random-effects meta-regressions were conducted by examining the association of the mean age of the study, the year of publication, and the risk of bias score of the study with prevalence rates. We also compared the prevalence rates of studies focusing on COP and all other studies. All analyses were repeated after excluding childhood-onset psychotic disorders. Publication bias was examined by calculating fail-safe *N* and observing funnel plots and Egger’s Regression Test.

## Results

### Search Results

**Figure 1.**
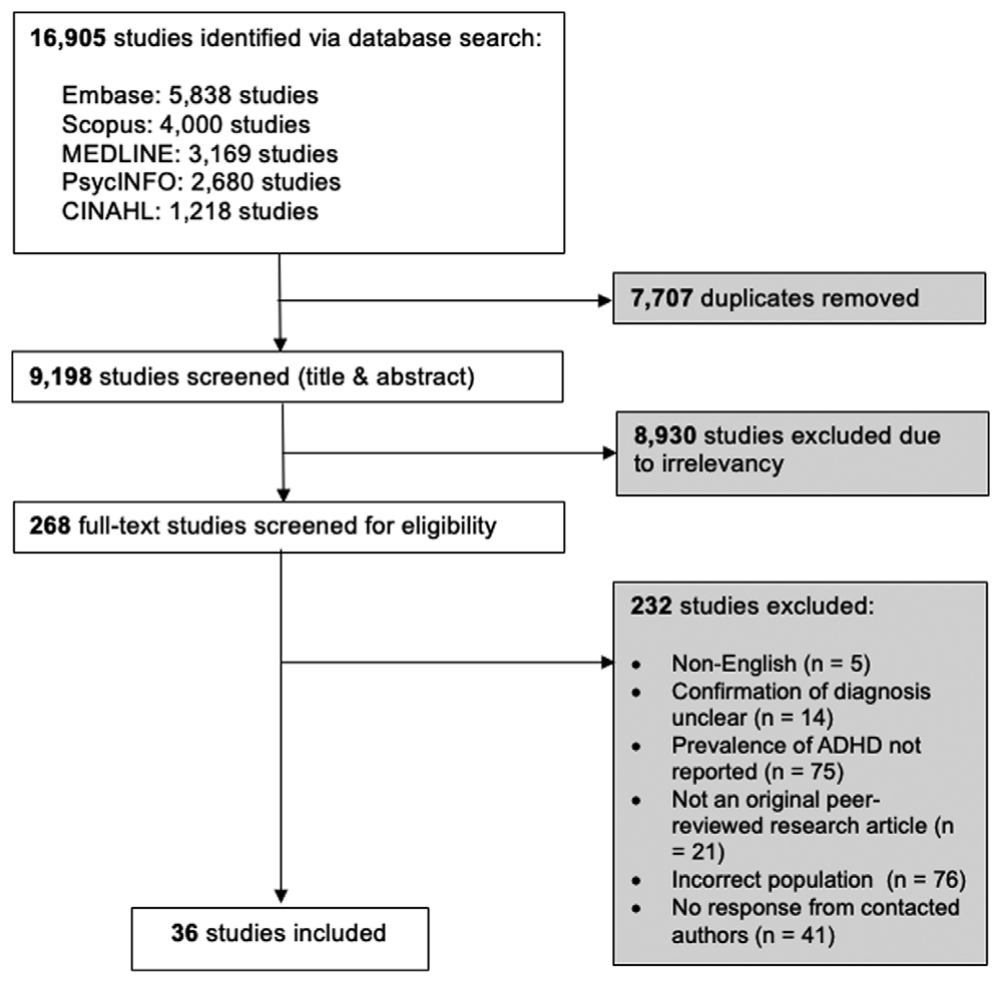
PRISMA Flow Diagram

The literature search yielded 9198 unique studies to assess for inclusion after the removal of duplicates. We included 36 studies after all phases of screening and contacting authors. Figure 1 presents the PRISMA flowchart.

### Description of Included Studies

Table 1 presents the characteristics of the included studies. A total of 30 726 participants were analyzed, with the reported proportion of females across studies ranging from 0% to 83%, and the mean age ranging from 9.87 to 42.50 years.

**Table 1.** Summary of Study Characteristics

Study	Country	Sample *N*	% Female	Age *M* (SD)	Study design	ADHD assessment mode	ADHD assessment name	ADHD assessment criteria	Psychotic disorder	Risk of bias score
Alavi et al. (2010)^61^	Iran	75	42.7	NR (NR)	Cross-sectional	Interview	K-SADS-PL	DSM-IV	Other psychotic disorder	7
Anckarsäter et al. (2007)^62^	Sweden	9	19.0	16.2 (1.8)	Cross-sectional	Interview	NR	DSM-IV	Other psychotic disorder	3
Andersen et al. (2013)^63^	Denmark	1667	35.6	26.3 (4.1)	Register	Interview	NR	ICD	Other psychotic disorder	6
Arican et al. (2019)^26^	UK	126	34.1	NR (NR)	Cross-sectional	Self-report	WURS, ASRS	DSM	Schizophrenia spectrum	6
Baeza et al. (2021)^64^	Spain	278	33.8	23.8 (NR)	Cohort	Interview	SCID, K-SADS	DSM-IV	Unclear	6
Brodsky et al. (2014)^29^	USA	40	35.0	10.3 (NR)	Cross-sectional	Mixed	K-SADS-PL	DSM-IV	Other psychotic disorder	6
Dalteg et al. (2014a)^65^	Finland	158	41.8	38.2 (NR)	Cross-sectional	Interview	NR	DSM-IV	Other psychotic disorder	3
Dalteg et al. (2014b)^65^	Mixed	247	0	38.4 (NR)	Cross-sectional	Interview	SCID	DSM-IV	Other psychotic disorder	3
Das et al. (2018)^66^	India	100	43.0	32.6 (NR)	Cross-sectional	Interview	MINI Plus 5.0	DSM-5	Other psychotic disorder	9
Donev et al. (2011)^67^	Germany	27	48.2	25.7 (7.6)	Cross-sectional	Mixed	WURS, Wender-Reimherr Interview, ADHD-HKS	ICD-10 & DSM-IV	Schizophrenia spectrum	3
Falcone et al. (2010)^68^	USA	102	34.3	14.8 (1.9)	Medical records	Unclear	NR	DSM-IV	Other psychotic disorder	4
Fond et al. (2022)^69^	France	916	26.2	32.0 (NR)	Cohort	Self-report	WURS-25	WURS-25	Schizophrenia spectrum	5
Frazier et al. (2022)^49^	USA	10	48.7	15.5 (0.5)	Cohort	Interview	MINI-KID	ICD-10 & DSM-5	Unclear	7
Gajwani et al. (2022)^48^	UK	6	79.0	NR (NR)	Cross-sectional	Self-report	ASRS	DSM-5	Other psychotic disorder	7
Hallerbäck et al. (2014)^70^	Sweden	41	39.0	28.9 (4.6)	Cross-sectional	Interview	SCID	DSM-IV	Schizophrenia spectrum	4
Hennig et al. (2017)^71^	UK	57	50.6	12.8 (0.2)	Cohort	Mixed	DAWBA	DSM-IV	Unclear	9
Hsu et al. (2019)^72^	Taiwan	401	50.12	15.8 (1.8)	Register	Unclear	NR	ICD-9-CM	Schizophrenia spectrum	6
Jerrell et al. (2016)^30^	USA	612	36.3	14.1 (3.0)	Register	Unclear	NR	ICD-9-CM	Other psychotic disorder	4
Kafali et al. (2019)^73^	Turkey	30	60	16.3 (1.8)	Cross-sectional	Interview	K-SADS-PL	DSM-IV	Schizophrenia spectrum	7
Maibing et al. (2015)^74^	Denmark	3,085	NR	NR (NR)	Register	Unclear	NR	ICD-10	Schizophrenia spectrum	5
Maydell et al. (2009)^31^	South Africa	12	50.0	NR (NR)	Cross-sectional	Unclear	NR	DSM-IV	Schizophrenia spectrum	5
Moe et al. (2022)^75^	USA	19 422	51.3	NR (NR)	Register	Unclear	NR	ICD-10	Other psychotic disorder	5
Moran et al. (2015)^76^	USA	199	33.7	34.1 (NR)	Cross-sectional	Self-report	Yes/No question	NR	Schizophrenia spectrum	4
Morgan & Cause (1999)^77^	USA	27	43.0	16.6 (NR)	Cross-sectional	Interview	DISC-2.1	DSM-III	Schizophrenia spectrum	5
Olashore et al. (2017)^50^	Botswana	35	39.5	12.4 (4.1)	Medical records	Unclear	NR	ICD-10	Unclear	3
Ordóñez et al. (2016)^78^	USA	133	45.9	9.9 (NR)	Cohort	Interview	K-SADS	NR	Schizophrenia spectrum	6
Paruk et al. (2022)^51^	South Africa	14	46.4	NR (NR)	Cross-sectional	Unclear	NR	DSM-5	Schizophrenia spectrum	7
Peralta et al. (2011)^79^	Spain	122	31.2	27.8 (8.4)	Cross-sectional	Interview	WURS	DSM-IV	Schizophrenia spectrum	6
Rho et al. (2015)^28^	Canada	179	27.9	23.6 (NR)	Cohort	Interview	CORS	DSM-IV	Other psychotic disorder	7
Ross et al. (2006)^80^	USA	83	27.7	9.9 (2.5)	Cross-sectional	Interview	K-SADS-PL	DSM-IV	Schizophrenia spectrum	6
Rubino et al. (2009)^81^	Italy	197	37.1	35.8 (11.1)	Cross-sectional	Interview	K-SADS	DSM-IV	Schizophrenia spectrum	7
Sanchez-Gistau et al. (2020)^27^	Spain	133	32.3	22.14 (5.3)	Cross-sectional	Interview	DIVA	DSM-IV	Other psychotic disorder	9
Srivastava et al. (2021)^82^	India	27	32.2	NR (NR)	Medical records	Interview	NR	DSM-IV	Unclear	4
Steyn et al. (2022)^52^	South Africa	8	83.4	34.4 (9.9)	Medical records	Unclear	NR	DSM-5	Schizophrenia spectrum	5
Strålin et al. (2019)^5^	Sweden	2,091	36.0	NR (NR)	Register	Unclear	NR	ICD-10	Other psychotic disorder	6
Syed et al. (2010)^6^	Ireland	57	56.4	42.5 (NR)	Cross-sectional	Self-report	ASRS	DSM-IV	Schizophrenia spectrum	4

Abbreviations: ADHD-HKS, 22-item self-rating scale (German); ASRS, Adult AHDD Self-Report Scale; CORS, circumstances of onset and relapse schedule; DAWBA, The Development and Well-Being Assessment; DISC, diagnostic interview schedule for children; DIVA, diagnostic interview for ADHD in adults; DSM, diagnostic statistical manual; ICD, International Classification of Diseases; K-SADS-PL, Kiddie Schedule for Affective Disorders and Schizophrenia Present and Lifetime Version; MINI, Mini International Neuropsychiatric Interview; MINI-KID, Mini International Neuropsychiatric Interview for Children and Adolescents; NR, not reported; SCID, Structured Clinical Interview for DSM; WURS, Wender Utah Rating Scale.

Most samples comprised both schizophrenia spectrum and non-schizophrenia psychotic disorders. Because of this, studies were considered to focus on 1 diagnostic type if 80% or more of the sample comprised either schizophrenia or non-schizophrenia psychotic disorders. In total, 20 (56%) studies focused on schizophrenia spectrum disorders, 11 (31%) non-schizophrenia spectrum psychotic disorders, and 5 (14%) studies did not provide clear information on the diagnosis. Six studies focused on COP.

Approximately, half of the included studies were cross-sectional (*n* = 20, 56%); 6 studies (17%) were register studies, 4 (11%) medical record studies, and 6 (17%) cohort studies. Most studies recruited participants from a clinical setting (*n *= 27, 75%). The majority of studies were conducted in either Europe (*n *= 17, 47%) or North America (*n *= 10, 28%), with the rest coming from Africa (*n *= 4, 11%), Asia (*n *= 3, 8%), and the Middle East (*n *= 1, 2%). One study sample was from multiple regions and there were no studies from Oceania or South America.

Regarding ADHD assessment, 17 (47%) used an interview schedule and 5 (13%) used a self-report scale. Four (11%) studies assessed ADHD using both interview and self-report measures. Ten (28%) studies did not clearly describe how ADHD was assessed; for these studies, frequency counts of ADHD were generally derived from public registers or medical records. An informant (eg, caregiver and/or teacher) was involved in the assessment of ADHD in 14 (39%) studies, while 10 (28%) had no informant involvement. For the remainder (12 studies [33%]), it was unclear whether an informant was involved or not. Most studies reported ADHD according to DSM criteria, with 4 (11%) using the DSM-5, 20 (56%) using the DSM-IV, and 1 (3%) using the DSM-III. Five (14%) studies used ICD-10 and 2 (6%) used ICD-9 criteria. Two (6%) studies used both DSM and ICD criteria. One study used the Wender Utah Rating Scale (WURS) and 2 did not report the criteria used.

Six studies reported the frequency counts of DSM-IV ADHD subtypes. The mean prevalence of the inattentive subtype was 15% (SD = 14.18, range = 0%-37%), and 10% (SD* *= 19.17, range 0%-49%) for the hyperactivity-impulsivity subtype. The mean prevalence for the combined subtype was 15% (SD* *= 17.16, range 4%-46%).

### Quality Appraisal

Supplementary Table S1 reports the mean, standard deviation, and range of scores for risk of bias criteria. Overall, the risk of bias total scores ranged from 3 to 9 out of 10 (*M* = 5.53, SD* *= 1.70), and 25.6% of ratings required resolution by a third independent rater. Most studies included sufficient information about the methods used to assess psychotic disorders and ADHD. The participation rate was reported in 8 studies^6,26,27,48,49,51,66,71^ and was the most poorly met criterion overall.

### Pooled Prevalence of ADHD in Psychotic Disorders

**Figure 2.**
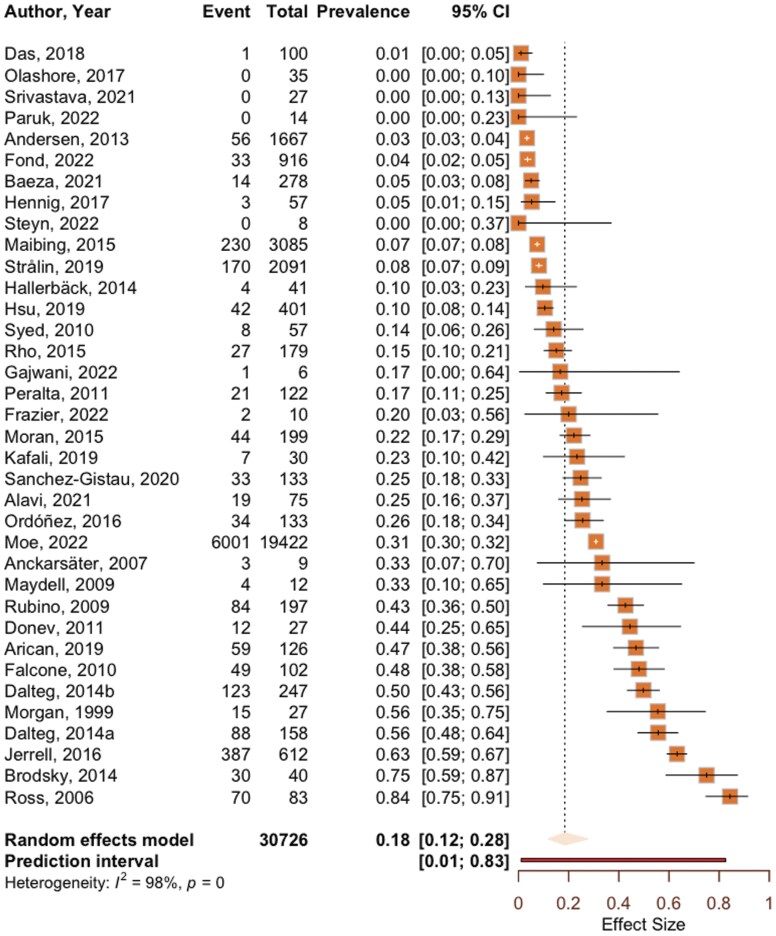
Forest Plot Showing the Pooled Prevalence of ADHD From the Generalized Mixed-Effects Model Analysis. For Each Study, “Event” Indicates the Number of Individuals With ADHD and “Total” Indicates the Sample Size. Individual Study Prevalence Estimates Are Shown With 95% Confidence Intervals

The pooled prevalence of ADHD in individuals with a psychotic disorder was 18.5% (95% CI [11.8%, 27.8%]). The between-study heterogeneity was high (*I*^2^ = 98.4% [95% CI 98.2%, 98.6%]) and the prediction interval indicated that the prevalence rate in future studies was between 1.1% and 82.6%. Figure 2 displays the forest plot of the pooled prevalence of ADHD in psychotic disorders for all studies. Repeating these analyses following the identification and exclusion of sixteen outliers resulted in a slight reduction of the pooled prevalence of 16.1% (see Supplementary Figure S1 for detail). Between-study heterogeneity was also reduced but remained high (*I*^2^ = 63.5% [95% CI 41.0%, 77.4%]) and the prediction interval ranged from 3.5% to 46.6%. Sensitivity analyses including studies with a sample size* *≥10 (*n* = 32 studies) and ≥20 (*n* = 30 studies) yielded similar results to analyses including all studies (see Supplementary Figures S2 and S3 for detail). There was no evidence of publication bias (fail-safe *N *= 22 426, Egger’s *t* = −1.2, *P* = .25 see Supplementary Figure S4 for funnel plot).

### Prevalence by Type of Psychotic Disorder

Subgroup analyses showed that the pooled prevalence of ADHD in people with schizophrenia spectrum disorders was 22.2% (95% CI [12.4%, 36.5%]) and 24.1% (95% CI [11.8%, 42.9%]) for people with non-schizophrenia psychotic disorders. The difference in pooled prevalence between people with schizophrenia spectrum and non-schizophrenia psychotic disorder samples was not significant (see Supplementary Table S2 and Supplementary Figure S5 for detail).

### Prevalence by Type of ADHD Assessment

Due to the low number of studies using DSM-III, ICD-9, mixed, or other ADHD diagnostic criteria, these studies were excluded from subgroup analyses. Overall, the difference in the pooled prevalence between studies using DSM-IV (27.1%, 95% CI [16.7%, 40.7%]), ICD-10 (7.1%, 95% CI [1.8%, 24.5%]), and DSM-5 criteria (1.6%, 95% CI [0.2%, 13.3%]) was statistically significant, *Q *= 20.4, df = 2, *P *< .001. Pairwise differences showed that studies using the DSM-IV had a significantly higher prevalence than those using the ICD-10, *z* = 1.8, *P* ≤ .01, 95% CI [0.4, 4.7] or the DSM-5, *z *= 4.1, *P *< .001, 95% CI [1.6, 4.7] (see Supplementary Table S3 and Supplementary Figure S6 for detail). There were no significant differences in prevalence rates between ADHD assessment types (ie, Interview; Self-report; Other; Unclear), *Q *= 1.4, df = 3, *P *= .71, nor between studies with and without informant involvement, *Q *= 3.2, df = 2, *P *= .20 (see Supplementary Figures S7 and S8 for detail).

### Prevalence by Region

Due to the low number of studies from outside Europe and North America, studies from Africa, Asia, and the Middle East were combined into 1 regional category. North America reported the highest prevalence (43.3%, 95% CI [25.8%, 62.7%]), followed by Europe (15.6%, 95% CI [9.1%, 25.5%]), and the rest of the world (4.2%, 95% CI [0.5%, 28.5%]) (see Supplementary Figure S9). Subgroup analyses revealed these differences were significant (*Q* = 14.1, df = 2, *P *< .001), with North America having a significantly higher prevalence than Europe, *z *= −3.1, *P* ≤ .01, 95% CI [−2.3, −0.5], and the Rest of the World, *z* = −2.8, *P* ≤ .01, 95% CI [−4.87, −0.86]. The difference between Europe and the Rest of the World was not significant, *z *= −1.4, *P = *.15, 95% CI [−3.4, 0.5].

### Prevalence by Study Characteristics

Cross-sectional studies reported the highest prevalence (29.8%, 95% CI [17.8%, 45.4%]), followed by register studies (14.4%, 95% CI [4.2%, 39.4%]), cohort studies (9.2%, 95% CI [3.8%, 20.8%]), and medical record studies (0.3%, 95% CI [0.0%, 99.8%]) (see Supplementary Figure S10). There was a significant difference in the pooled prevalence of ADHD in psychotic disorders between the types of study designs, *Q* = 10.1, df = 3, *P* ≤ .05. Pairwise comparisons showed that cross-sectional studies reported significantly higher prevalence rates than cohort studies, *z *= −2.9, *P *< .01, 95% CI [−2.4, −0.5]. Pairwise differences between other types of study design were not statistically significant (see Supplementary Table S5 for detail).

The pooled prevalence in study samples from clinical settings was 15.8% (95% CI [8.7%, 27.2%]) and 27.2% in nonclinical settings (95% CI [14.0%, 46.1%]) (see Supplementary Figure S11 for forest plot). The difference between these prevalence rates was not statistically significant, *Q* = 2.0, df = 1, *P *= .16.

**Figure 3.**
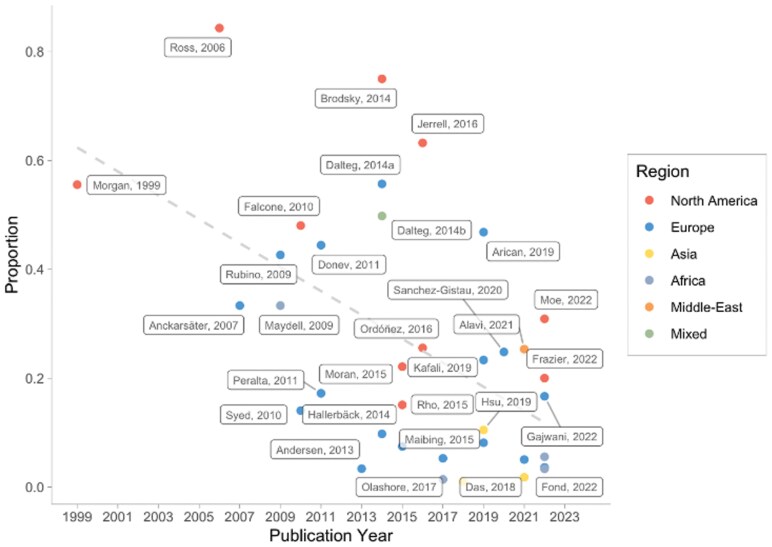
Meta-regression Results for Publication Year With the Geographical Region Indicated in Color-Coded Circles. Overall, the Prevalence Rates of ADHD Decrease as the Publication Year Increases. Studies Using North American Samples Tended to be Published Earlier Than Samples From Other Geographical Regions

Based on meta-regressions, the publication year (*b* = –0.2, *P *< .01, 95% CI [–0.2, –0.1]) had a significant association with ADHD prevalence in psychotic disorders, with more recent studies reporting lower rates of ADHD (see Figure 3). Mean age (*b* = –0.04, *P = *.24, 95% CI [–0.10, 0.03]) and risk of bias (*b* = –0.2, *P = *.18, 95% CI [–0.5, 0.1]) did not have a significant association with the prevalence of ADHD in psychotic disorders (see Supplementary Figures S12 and S13 for detail).

### Prevalence in Childhood-Onset Psychotic Disorders

The pooled prevalence of ADHD in COP samples was 52.3% (95% CI [25.4%, 78.0%]) and 14.3% (95% CI [8.9%, 22.2%]) for all other studies, which was a significant difference, *Q* = 12.9, df = 1, *P < *.001 (see Supplementary Figure S14 for detail).

After excluding COP samples, subgroup analyses revealed that prevalence rates based on DSM-IV diagnoses remained significantly higher than those based on ICD-10, *z* = 2.1, *P* ≤ .05, 95% CI [0.1, 2.5] and DSM-5, *z* = 2.8, *P* = <.01, 95% CI [1.3, 2.5] (see Supplementary Figure S15). Similarly, prevalence rates in North America remained significantly higher than those observed in Europe, *z* = 2.1, *P* ≤ .05, 95% CI [0.1, 1.7] and the rest of the world, *z* = 2.3, *P* ≤ .05, 95% CI [0.6, 6.3] (see Supplementary Figure S16). Cross-sectional studies continued to exhibit significantly higher prevalence rates compared to cohort studies, *z* = 3.3, *P* ≤ .01, 95% CI [0.6, 2.4], and were now also significantly higher than those reported in register-based studies, *z* = 2.3, *P* ≤ .05, 95% CI [0.2, 2.1] (see Supplementary Table S5 and Supplementary Figure S17 for detail). Additionally, the publication year significantly influenced ADHD prevalence, with more recent studies reporting lower rates, *b* = –0.1, *P *< .01, 95% CI [–0.2, –0.04]. The differences in pooled prevalence rates between schizophrenia spectrum and non-schizophrenia psychotic disorders, between diagnoses with and without informant involvement, between types of ADHD assessments, and between various clinical settings remained statistically nonsignificant (see Supplementary Figures S18–S21). Meta-regressions did not demonstrate any significant effects of mean age or risk of bias score on the prevalence rates (see Supplementary Figures S22–S24).

## Discussion

To our knowledge, this is the first study to comprehensively synthesize the published prevalence rates of ADHD in psychotic disorders. We included 36 studies comprising a total of 30 726 participants. Overall, the pooled lifetime prevalence of ADHD in psychotic disorders was 18.49%, a figure that is higher than the prevalence in the general population, which ranges from 5% to 8% in children and adolescents, and 2%-6% in adults.^1^ This pattern of relatively higher prevalence is consistent with that found in other mental disorders, including affective and anxiety disorders.^4,83^ However, consistent with the literature and the reported prevalence of ADHD in the general population, we observed wide variability in prevalence rates,^2,3^ suggesting that the overall pooled prevalence should be interpreted with caution.

There are several reasons that may be associated with wide variation in the reported prevalence of ADHD in psychotic disorders. One reason is that samples using DSM-IV ADHD diagnostic criteria reported higher prevalence rates than those using ICD-10 criteria. This is consistent with previous general population studies, which have observed higher ADHD prevalence rates with DSM-IV compared to ICD-10.^3,32^ This may be because ICD-10 classified ADHD under Hyperkinetic Disorders, which required individuals to exhibit both inattentive and hyperactive/impulsive symptoms for an ADHD diagnosis.^84^ As demonstrated by a mean prevalence of 15% in 6 of our included studies, individuals with psychotic disorders often present with the ADHD inattentive subtype. The DSM-IV criteria may have been more inclusive of these individuals within the psychotic disorder population. These findings suggest that clinicians should carefully consider the diagnostic criteria they employ when assessing ADHD in individuals with psychotic disorders. Notably, most studies in our analysis utilized DSM-IV and ICD-10 criteria; however, the more recent ICD-11 has redefined its criteria to better align with DSM-5, now including predominantly inattentive and predominantly hyperactive-impulsive presentations.^43^ As DSM-5 and ICD-11 become more widely adopted in future studies, it will be important to examine the impact of their use on ADHD prevalence rates in psychotic disorder populations.

Another reason for the wide variability in prevalence rates appears to be the markedly higher prevalence of ADHD in COP compared to adolescent- and adult-onset psychotic disorders. This finding lends support to the notion that neurodevelopmental conditions are more prevalent in COP compared to adolescent- and adult-onset psychotic disorders.^85,86^ For example, Jerrell et al. indicated that individuals who developed a psychotic disorder before the age of 12 were twice as likely as those with adolescent-onset psychosis to experience neurodevelopmental difficulties, including speech, language, and educational challenges, as well as ADHD.^30^ From an etiological standpoint, our findings are notable as it may suggest that COP represents a distinct entity, which has shown to involve more severe clinical and functional outcomes than psychotic disorders developed during adolescence and adulthood.^87^ For example, COP is associated with more severe positive and negative symptoms, poorer school performance, lower likelihood of employment, and longer duration of untreated psychosis compared to adolescent-onset psychotic disorder.^85,86,88,89^ Cognitive impairments, such as intellectual functioning, learning and memory, and perceptuomotor skills, are also more pronounced in this population.^88^ Furthermore, the higher prevalence of ADHD in COP samples may indicate potential iatrogenic effects, whereby medications used to treat ADHD during childhood may be associated with the subsequent onset of psychosis.^90^ Overall, the higher prevalence of ADHD in COP suggests that ADHD, alongside other neurodevelopmental conditions, may play a significant role in the adverse outcomes associated with COP.

We also found that cross-sectional studies reported relatively higher prevalence rates than longitudinal cohort studies. Previous research indicates that poor functional outcomes, severe mental health symptoms, and significant cognitive impairments, characteristics commonly associated with comorbid ADHD and psychotic disorders, are linked to higher rates of loss to follow-up in longitudinal studies.^91,92^ Consequently, individuals who are later diagnosed with a psychotic disorder, and thus present with comorbid ADHD, may show a lower adherence to follow-up, potentially resulting in underestimated prevalence rates of these conditions in longitudinal research.^93^ Furthermore, at a trend level, register-based studies tended to report lower prevalence rates, which is a common finding in many prevalence meta-analyses.^58^ Researchers should consider these factors when interpreting findings regarding the prevalence of ADHD in psychotic disorders.

Regional differences in ADHD prevalence rates were also found to account for the variation observed in our study. Specifically, North American samples exhibited a higher prevalence of ADHD in psychotic disorders compared to European and other international samples. This finding is consistent with reports indicating elevated ADHD prevalence within the general population in North America.^33^ Such regional disparities may reflect sociocultural factors, including differential access to healthcare, variations in diagnostic incentives, and differences in professional training, which vary by region.^34–36,94^ However, the precise impact of these factors remains speculative due to limited available data. Furthermore, methodological differences in ADHD assessment may be associated with these variations. A previous meta-analysis of general population studies did not reveal significant regional differences in prevalence, suggesting that diagnostic criteria, study design, and study settings are more influential in driving prevalence rate disparities.^3^ For instance, the DSM has been predominantly employed in North American studies, whereas the ICD is more commonly used in European studies.^34^ Our findings indicate that many North American studies are cross-sectional, published earlier, and often involve COP samples. Despite excluding COP samples, regional differences persisted, suggesting that this factor alone does not fully account for the observed variations. Overall, while our study identifies notable geographical differences, these should be interpreted with caution, given the potential overlap of other factors observed.

Our study identified a negative association between the year of publication and the reported prevalence of ADHD in psychotic disorders, with more recent studies indicating lower prevalence rates. This finding stands in contrast to the increasing prevalence of ADHD in the general population over recent years.^95^ One potential explanation is that earlier studies, being fewer in number and potentially underpowered, may reflect a bias towards overstated prevalence rates.^96^ As the body of literature has expanded in recent years, often with more rigorous methodologies, these estimates may reflect a more accurate estimation of the prevalence rate.^96^ Additionally, a substantial proportion of the earlier studies originated from North America, a region with a long-standing emphasis on ADHD in psychotic disorders. One review highlighted that ADHD research was rooted in the United States and, since the 1990s, has expanded globally, with a higher concentration of prevalence studies in the United States and Europe until the 2010s.^34^ The inclusion of more recent studies from other regions, which tend to report lower prevalence rates, may also be associated with the observed decline. Consequently, the negative association with the year of publication may be indicative of these regional trends.

Despite significant variability in prevalence estimates, ADHD appears to be more common in psychotic disorders than in the general population, highlighting the need for clinical attention. For example, routine screening and thorough assessment of ADHD in individuals with psychotic disorders are crucial for accurate clinical formulation, disentangling potential diagnostic overlap, and tailoring treatment to support symptom and functional recovery. While the higher prevalence of ADHD in psychotic disorders aligns with patterns observed in other mental disorders, such as affective and anxiety disorders, the mechanisms driving this association, such as dopaminergic dysregulation^13–15^ and the use of psychostimulants,^19–21^ may differ from those in other conditions.^4,83^ Clinically, individual formulations may also consider important contextual factors, such as how ADHD-related social exclusion may contribute to psychosis risk.^97,98^ Therefore, our findings emphasize the importance of continued research into the shared and distinct etiological pathways of ADHD and psychosis, which could inform more effective treatment strategies. Overall, these findings build on previous studies with etiological implications, such as those indicating that individuals with ADHD are at higher risk of developing a psychotic disorder.^11^ This study quantifies the prevalence of ADHD within psychotic disorders and examines variations in prevalence rates, offering important insights for clinical awareness.

The current findings must be interpreted considering several limitations. First, we included only studies published in English, which may introduce a language bias and partially explain the greater number of studies from North America and Europe compared to regions such as Africa, the Middle East, and Asia. Second, it was challenging to delineate between schizophrenia spectrum and non-schizophrenia psychotic disorders at the study level. Most samples included both types of disorders, making it uncertain whether all frequency counts were accurately allocated to individuals with schizophrenia spectrum disorders or non-schizophrenia psychotic disorders. Third, we could not examine the effects of sex on prevalence rates, as the included studies did not provide frequency counts or prevalence data by sex. This is an important limitation as ADHD may present differently between males and females.^99^ Fourth, it was difficult to ascertain the exact methods used for ADHD assessment, as this was not reported in detail in several studies. Consequently, performing subgroup analyses based on variables related to ADHD assessment was potentially imprecise. This is critical as methodological differences have been cited as a significant reason for the large variation in ADHD prevalence rates in previous studies. Therefore, future studies investigating the relationship between ADHD and psychotic disorders should provide detailed descriptions of the assessment methods used for ADHD. This includes specifying whether an interview was conducted, the involvement and type of informants, the personnel involved in conducting assessments, and any other corroborative information used to confirm ADHD. Several clinical practice guidelines recommend including these elements in ADHD diagnostic assessments.^100^ Fifth, it is possible that prevalence rates reflect diagnostic overlap during the CHR state for psychosis, where individuals with subthreshold psychotic symptoms may be misidentified as having ADHD before transitioning to full-threshold psychosis.^25^ However, examining prevalence rates in CHR samples was beyond the scope of this study. Sixth, while our findings suggest a higher prevalence of ADHD in psychotic disorders compared to the general population, readers should interpret this inference cautiously. The differing life expectancies between individuals with psychotic disorders and those without^101^ may skew lifetime prevalence rates, a limitation inherent in the available evidence. Finally, there was a significant overlap between the factors identified in explaining the variation in prevalence rates, suggesting potential confounding in the subgroup analyses. However, due to the small sample sizes within each subgroup, we were unable to control for these confounding factors. Consequently, the association of each factor should be interpreted with caution.

## Conclusion

The prevalence of ADHD among individuals with psychotic disorders was found to be notably higher (18%) compared to that in the general population (5%-8%). This highlights a need for greater clinical attention to this comorbidity. The reported prevalence rates, however, exhibit considerable variation, with substantial heterogeneity observed across studies. This variability may be attributable to several factors, including differences in diagnostic criteria for ADHD, the presence of COP, year of publication, and geographical region. Our findings suggest that multiple factors may be associated with the wide variability in reported ADHD prevalence among individuals with psychotic disorders. Clinicians should consider these factors when diagnosing ADHD as a comorbidity in individuals with psychotic disorders. To better understand the variability in prevalence rates, future research should explore the significant overlap among these factors, potentially by employing more rigorous and standardized ADHD assessment protocols.

## Supplementary Material

Supplementary material is available at https://academic.oup.com/schizophreniabulletin.

sbae228_suppl_Supplementary_Tables_S1-S5_Figures_S1-S24
