# Supplementary material for: The Prevalence of Attention Deficit Hyperactivity Disorder in Psychotic Disorders: Systematic Review and Meta-analysis
Source: Schizophr Bull. 2025 Jan 13;51(6):1514–28. doi: 10.1093/schbul/sbae228 (PMC12599807; doi:10.1093/schbul/sbae228)
Supplement: sbae228_suppl_Supplementary_Tables_S1-S5_Figures_S1-S24 [file sbae228_suppl_supplementary_tables_s1-s5_figures_s1-s24.html]

Supplementary Tables and Figures


# Supplementary Tables and Figures

#### Nicholas Cheng

The supplementary tables and figures for the Cheng et al. manuscript,
*“The prevalence of ADHD in psychotic disorders: Systematic review
and meta-analysis”*, submitted to *Schizophrenia Bulletin*,
are displayed below.

Version 14-08-2024

---

# Supplementary Tables

---

##### Table S1.

**Mean, standard deviation, and range for risk of bias
criteria**

| Risk of Bias Criteria | Mean | Standard Deviation | Minimum | Maximum |
| --- | --- | --- | --- | --- |
| Psychotic Disorder Assessment | 1.50 | 0.56 | 0 | 2 |
| Study Setting | 1.25 | 0.44 | 1 | 2 |
| Inclusion/Exclusion Criteria | 1.03 | 0.81 | 0 | 2 |
| Participation Rate | 0.33 | 0.68 | 0 | 2 |
| ADHD Assessment | 1.42 | 0.50 | 1 | 2 |
| Total Score | 5.53 | 1.70 | 3 | 9 |

---

##### Table S2.

**Pairwise comparisons for subgroup analysis (Psychotic
Disorder Type)**

| Psychotic Disorder Type | Difference | SE | z-score | p-value | 95% CI (Lower Bound) | 95% CI (Upper Bound) |
| --- | --- | --- | --- | --- | --- | --- |
| Other Psychotic Disorder vs Schizophrenia Spectrum Disorder | 0.11 | 0.51 | 0.21 | 0.83 | -0.90 | 1.11 |
| Other Psychotic Disorder vs Unclear | 1.87 | 0.45 | 4.12 | 0.00 | 0.98 | 2.76 |
| Schizophrenia Spectrum Disorder vs Unclear | 1.76 | 0.41 | 4.31 | 0.00 | 0.96 | 2.56 |

---

##### Table S3.

**Pairwise comparisons for subgroup analysis (ADHD Diagnostic
Criteria)**

| Diagnostic Criteria | Difference | SE | z-score | p-value | 95% CI (Lower Bound) | 95% CI (Upper Bound) |
| --- | --- | --- | --- | --- | --- | --- |
| DSM-IV vs DSM-V | 3.15 | 0.77 | 4.09 | 0.00 | 1.64 | 4.66 |
| DSM-IV vs ICD-10 | 1.58 | 0.60 | 2.64 | 0.01 | 0.41 | 2.75 |
| DSM-V vs ICD-10 | -1.57 | 0.88 | -1.78 | 0.07 | -3.30 | 0.16 |

---

##### Table S4.

**Pairwise comparisons for subgroup analysis (Study
Design)**

| Study Design | Difference | SE | z-score | p-value | 95% CI (Lower Bound) | 95% CI (Upper Bound) |
| --- | --- | --- | --- | --- | --- | --- |
| Register vs Medical Records | 3.99 | 3.78 | 1.06 | 0.29 | -3.42 | 11.40 |
| Register vs Cohort | 0.51 | 0.64 | 0.79 | 0.43 | -0.76 | 1.77 |
| Register vs Cross-Sectional | -0.93 | 0.62 | -1.50 | 0.13 | -2.14 | 0.28 |
| Medical Records vs Cohort | -3.49 | 3.76 | -0.93 | 0.35 | -10.86 | 3.89 |
| Medical Records vs Cross-Sectional | -4.92 | 3.76 | -1.31 | 0.19 | -12.28 | 2.44 |
| Cohort vs Cross-Sectional | -1.43 | 0.49 | -2.93 | 0.00 | -2.39 | -0.47 |

---

##### Table S5.

**Pairwise comparisons for subgroup analysis (Study Design)
with COP samples excluded**

| Study Design | Difference | SE | z-score | p-value | 95% CI (Lower Bound) | 95% CI (Upper Bound) |
| --- | --- | --- | --- | --- | --- | --- |
| Register vs Medical Records | 3.53 | 3.76 | 0.94 | 0.35 | -3.84 | 10.90 |
| Register vs Cohort | 0.36 | 0.50 | 0.72 | 0.47 | -0.61 | 1.33 |
| Register vs Cross-Sectional | -1.12 | 0.48 | -2.32 | 0.02 | -2.06 | -0.18 |
| Medical Records vs Cohort | -3.17 | 3.76 | -0.84 | 0.40 | -10.53 | 4.19 |
| Medical Records vs Cross-Sectional | -4.65 | 3.75 | -1.24 | 0.22 | -12.00 | 2.71 |
| Cohort vs Cross-Sectional | -1.48 | 0.45 | -3.31 | 0.00 | -2.35 | -0.60 |

---

##### Table S6.

**Pairwise comparisons for subgroup analysis (Informant
Involvement) with COP samples excluded**

| Informant Involvement | Difference | SE | z-score | p-value | 95% CI (Lower Bound) | 95% CI (Upper Bound) |
| --- | --- | --- | --- | --- | --- | --- |
| No Informant vs Informant Involvement | 1.04 | 0.58 | 1.78 | 0.08 | -0.11 | 2.19 |
| No Informant vs Unclear | 1.43 | 0.58 | 2.46 | 0.01 | 0.29 | 2.56 |
| Informant Involvement vs Unclear | 0.39 | 0.65 | 0.60 | 0.55 | -0.88 | 1.65 |

---

# Supplementary Figures

---

##### Figure S1.

**Forest plot of pooled prevalence after excluding
outliers**

---

##### Figure S2.

**Forest plot of pooled prevalence after excluding studies with
a sample size of <10**

---

##### Figure S3.

**Forest plot of pooled prevalence after excluding studies with
a sample size of <20**

---

##### Figure S4.

**Funnel plot**

---

##### Figure S5.

**Forest plot of subgroup analysis (Psychotic Disorder
Type)**

---

##### Figure S6.

**Forest plot of subgroup analysis (ADHD Diagnostic
Criteria)**

---

##### Figure S7.

**Forest plot of subgroup analysis (ADHD Assessment
Type)**

---

##### Figure S8.

**Forest plot of subgroup analysis (Informant
Involvement)**

---

##### Figure S9.

**Forest plot of subgroup analysis (Region)**

---

##### Figure S10.

**Forest plot of subgroup analysis (Study Design)**

---

##### Figure S11.

**Forest plot of subgroup analysis (Study Setting)**

---

##### Figure S12.

**Bubble plot of meta-regression analysis (Mean
Age)**

---

##### Figure S13.

**Bubble plot of meta-regression analysis (Risk of Bias Total
Score)**

---

##### Figure S14.

**Forest plot of subgroup analysis (Childhood-Onset Psychotic
Disorder)**

---

##### Figure S15.

**Forest plot of subgroup analysis (ADHD Diagnostic Criteria),
after excluding COP samples**

---

##### Figure S16.

**Forest plot of subgroup analysis (Region)**

---

##### Figure S17.

**Forest plot of subgroup analysis (Study Design), after
excluding COP samples**

---

##### Figure S18.

**Forest plot of subgroup analysis (Psychotic Disorder Type),
after excluding COP samples**

---

##### Figure S19.

**Forest plot of subgroup analysis (Informant Involvement),
after excluding COP samples**

---

##### Figure S20.

**Forest plot of subgroup analysis (ADHD Assessment Type),
after excluding COP samples**

---

##### Figure S21.

**Forest plot of subgroup analysis (Study Setting), after
excluding COP samples**

---

##### Figure S22.

**Bubble plot of meta-regression analysis (Mean Age), after
excluding COP samples**

---

##### Figure S23.

**Bubble plot of meta-regression analysis (Risk of Bias Total
Score), after excluding COP samples**

---

##### Figure S24.

**Bubble plot of meta-regression analysis (Publication Year),
after excluding COP samples**
